# Prevalence and determinants of nutritional status among women and children in Pakistan

**DOI:** 10.1186/s12889-022-13059-2

**Published:** 2022-04-15

**Authors:** Hanumant Waghmare, Shekhar Chauhan, Santosh Kumar Sharma

**Affiliations:** International Institute for Population Sciences, Mumbai, India

**Keywords:** Body-mass index, Stunting, Wasting, Underweight, Pakistan

## Abstract

**Background:**

Nutrition has been a low-priority area in Pakistan, with low visibility from the political leadership. Despite various efforts, Pakistan has been reported to have one of the highest prevalences of child and women malnutrition compared to other developing counties. Therefore, this study intends to examine the prevalence and determinants of nutritional status of women and children in Pakistan.

**Methods:**

The present study uses the Demographic Health Survey (DHS) data from Pakistan 2012–13 (PDHS-3). The nutritional status of women was examined through Body-Mass Index (Underweight, normal, overweight, & obese), and that of children was examined through stunting (severe and moderate), wasting (severe, moderate, overweight), and underweight (severe, moderate, overweight). Descriptive statistics and bivariate analysis have been used along with multinomial logistic regression.

**Results:**

A higher proportion of children in rural areas were severely stunted (19.6% vs. 12.5%), severe wasted (2.4% vs. 2.2%), and severe underweight (9.4% vs. 6%) than their urban counterparts. A higher proportion of rural women (9.5% vs. 5.5%) were underweight than urban women, whereas a higher proportion of urban women were obese (24.3% vs. 19.0%) than rural women. The odds of severe stunting (OR = 0.24; C.I. = 0.15–0.37), severe underweight (OR = 0.11; C.I. = 0.05–0.22) were lower among children from the richest wealth quintile than their poorest counterparts. The Relative Risk Ratio (RRR) of being overweight (RRR = 3.7; C.I. = 2.47–5.54) and Obese (RRR = 4.35; C.I. = 2.67–7.07) than normal BMI were higher among women from richest wealth quintile than women belonged to poorest wealth quintile.

**Conclusion:**

This study has highlighted determinants associated with maternal and child nutritional status, whereby the child’s nutritional status was measured by stunting, wasting, and underweight, and BMI measured the mother’s nutritional status. The main risk factors for a child’s poor nutritional status include low household wealth, urban residence, and mother’s educational status. Similarly, the main risk factors for women’s poor nutritional status include increasing the women’s age, educational status, rural residence, and household wealth. Poor households should be provided special attention to improve the nutritional status among women and children in poor households.

## Background

Child undernutrition is a significant public health concern for children under five years of age in underdeveloped nations, including Pakistan. Malnutrition is produced by numerous interconnected causes and has short- and long-term adverse health consequences [1, 2]. According to the 2011 National Nutrition Survey of Pakistan, 31% of children under the age of five are underweight, whereas recent research in Pakistan found that the current incidence of underweight children is 29% [1, 3]. Pakistan was identified as one of the seven countries that accounted for about one-third of the world’s undernourished population, along with Bangladesh, China, Congo, Ethiopia, India, and Indonesia [4].

Undernutrition not only leads to illness and mortality among children, but it also impairs their physical and cognitive development, their ability to perform academically, and their ability to work later in life [5–7]. Consequently, undernutrition is one of the most pressing issues due to its long-term and adverse effects [8]. Several studies reported that inadequate nutrition is the significant risk factor for child malnutrition [9–11]. Studies have found that there are multiple factors associated with child undernutrition, such as low birth weight, mother’s education, mother’s body mass index (BMI), sex of the child, birth order, poor exclusive breastfeeding, poor sanitation practices, poverty, dietary diversity, and social inequalities [9, 10, 12–16]. Some studies have shown that individual and community level factors are responsible for childhood undernutrition [12, 13].

According to Cuming and Cairncross (2016), water, sanitation, and hygiene are recognized as significant risk factors for the health of infants and young children, where stunting is highly concentrated [17]. In Uganda, it was found that children belonging to lower socio-economic strata are more likely to be undernourished due to their higher vulnerability to food insecurity [18].

Child undernutrition can be attributed to many factors, including the mother’s nutritional status [19]. Children born to malnourished mothers are more likely to be underweight, which can run in families [20]. Several studies have documented a significant relationship between mother’s poor nutritional status and various pregnancy outcomes such as low birth weight, susceptibility to infections, and growth-challenged and developmentally delayed children [21]. Several factors affect the mother’s nutrition: high fertility, poor diet, low socio-economic status, cultural factors, fertility preferences, and closed birth interval. A high fertility rate combined with a lack of birth spacing results in a continuous cycle of pregnancy and breastfeeding, depleting a mother’s nutritional reserves. As a result, a woman’s parity and birth spacing significantly influence the child’s survival prospects [22]. A short inter-pregnancy gap does not provide enough time for the mother to recuperate from the delivery process and restore her reserves of nutrients used during pregnancy, especially when she is undernourished [23].

Pakistan is distinct in its own right, with varying levels of development in terms of nutrition policies and programs and different methods to improve nutrition services for women and children. The country offers a wealth of information about what is being done to enhance women’s and children’s nutrition and what needs to be done [3]. The double burden of malnutrition is becoming a growing problem in Pakistan, with overweight women outnumbering underweight women [3]. On the other hand, nutrition remains an unfinished business for Pakistan’s mothers and children [3]. As the country himself acknowledges, much more must be done to enable women to avoid the perils of undernutrition and, indeed, the growing risk of overweight/obesity [3]. In light of the above discussions, this study intends to examine the prevalence and determinants of nutritional status of women and children in Pakistan.

In their study, Khan et al. (2019) have already discussed the determinants of stunting, wasting, and underweight among children below five years of age using the same dataset. However, this study differs in many aspects from Khan et al. [1]. The study by Khan et al. categorized malnutrition as stunting, wasting, and underweight [1]. This study categorized these three categories of malnutrition as severe and moderate, thereby categorizing stunting as severe stinting and moderate stunting, and so on. Furthermore, Khan et al. only discussed the determinants of stunting, wasting, and underweight among children below five years of age; however, this study also included mothers exploring the risk of BMI among women aged 15–49 years of age. Furthermore, both the studies differ in terms of covariates.

## Data and methods

### Data

The present study uses the Demographic Health Survey (DHS) data from Pakistan 2012–13 (PDHS-3). The overall objective of the 2012–13 PDHS was to gather data on high standards of fertility, preference and use contraception and maternal and child health, infant mortality levels, vaccination, mother and child’s nutrition, and awareness of HIV/AIDS, tuberculosis, and other diseases. The overarching objective was to provide the information necessary for evidence-based planning by health and family planning programs and guide program managers and policymakers to plan and carry out future interventions successfully. PDHS has adopted a two-stage stratified sampling design. The rural and urban population of all four provinces of Pakistan (Punjab, Sindh, Khyber Pakhtunkhwa, and Baluchistan) and regions of Gilgit Baltistan and Islamabad Capital Territory (ICT) were included in the survey. The details of the sampling design were published in a report by Pakistan Demographic Health Survey [24].

The survey collected information from 12,943 households with 13,558 eligible women (15–49) and 3134 men (15–54). The anthropometric parameters (height and weight) of women and children were also collected in the survey. In Pakistan, PDHS measured all children aged less than five years; however, for women aged 15–49 years, height and weight were measured in every third household selected for male interview. Eligible children included were born within the five years preceding the survey and had a valid record of dates of birth. Measurements of children were recorded for both height (in centimeter) and weight (in kilograms) using the measuring boards of Shorr productions and the digital SECA scales, respectively. Out of 3466 eligible children born to 13,588 ever-married women aged 15–49 years, anthropometric information was available for 3071 children aged 0–59 months.

### Variable description

#### Dependent variable

 The present study is divided into two sections: nutritional status of women aged 15–49 years and nutritional status of children aged less than five years in Pakistan. The nutritional status of women is examined through the body mass index (BMI). It is categorized into four categories: Under-weight, Normal weight, Overweight and Obese. However, the nutritional status of children is examined through Z-scores of three parameters: Stunting, Wasting, and Under-weight & Over-weight for age. Stunting is categorized into two categories (Severe and Moderate); Wasting is categorized into three categories (Severe, Moderate, Over-weight); Weight for age is categorized into three categories (Severe underweight, Moderate underweight, and Overweight).

Women nutritional status: Cut-off limit for BMI (Weight for Height:

- Underweight: BMI < 18.5 kg/m^2^, Normal weight: BMI > =18.5 & < 24.9 kg/m^2^, Overweight: BMI > =30.0 & < 29.9 kg/m^2^, Obese: BMI > =30.0 kg/m^2^

Children nutritional status: Cut-off limit for Stunting (Height-for-Age):

- Severely Stunted: Z-score < − 3.0 SD below mean

- Moderately Stunted: Z-score < − 2.0SD below mean

Cut-off limit for Wasting (Weight-for-Height):

- Severely Wasted: Z-score < − 3.0 SD below mean

- Moderately Wasted: Z-score < − 2.0 SD below mean

- Overweight: Z-score > + 2.0 SD below mean

Cut-off limit for Weight-for-age:

- Severely underweight: Z-score < − 3.0 SD below mean

- Moderately underweight: Z-score < − 2.0 SD below mean

- Overweight: Z-score > + 2.0 SD below mean

#### Independent variables

 Women and children’s socioeconomic and demographic characteristics are considered to understand the nutritional status by selected background characteristics. The selected socio-economic and demographic characteristics to include: the place of residence (Rural, Urban), religion (Hindu, Muslim, Others), age (continuous), marital status (Married, Unmarried, Others), educational attainment, working status (Yes, No), Source of drinking water, type of cooking fuel, type of toilet facility, and wealth index. Apart from the variables mentioned above, the number of children and women’s dietary patterns (food composition) are considered. The number of siblings, sex, breastfeeding pattern, immunization, and dietary pattern are considered for children.

## Methods

Descriptive statistics and bivariate analysis have been used to understand the nutritional status among women aged 15–49 and children aged 0–59 months in Pakistan.

The multinomial logistic regression model was used to determine factors associated with Body Mass Index among women. This allowed us to assess the independent effect of background characteristics in assessing the prevalence of BMI. Multinomial logistic regression is an expansion of logistic regression in which one equation is set up for each logit relative to the reference outcome. BMI consists of four categories: normal, underweight, overweight, and obese. For a dependent variable with four categories, this requires the estimation of three equations, one for each category relative to the reference category (not related), to describe the relationship between the dependent and the independents variables:

1 $$\ln\ \left[\left\{P\left({Y}_i=2\right)|{X}_i\right\}/\left\{P\left({Y}_i=1\right)|{X}_i\right\}\right]={\alpha}_2+{\beta_1}^2{X}_1\dots \kern0.5em {\beta_k}^2{X}_{ik}$$

2 $$\ln\ \left[\left\{P\left({Y}_i=3\right)|{X}_i\right\}/\left\{P\left({Y}_i=1\right)|{X}_i\right\}\right]={\alpha}_3+{\beta_1}^3{X}_1\dots \kern0.5em {\beta_k}^3{X}_{ik}$$

3 $$\ln\ \left[\left\{P\left({Y}_i=4\right)|{X}_i\right\}/\left\{P\left({Y}_i=1\right)|{X}_i\right\}\right]={\alpha}_4+{\beta_1}^4{X}_1\dots \kern0.5em {\beta_k}^4{X}_{ik}$$

Where α_2_, α_3_, and α_4_ are the intercepts for the category underweight, overweight, and obese, respectively, and *β*_*k*_^2^, *β*_*k*_^3^, and *β*_*k*_^4^ are the slope coefficient of the *X*_*i*_ variables for respective category of the dependent variable.

We also used binary logistic regression to determine the factors associated with severe and moderate stunting, severe and moderate wasting, and severe and moderate underweight among children aged 0–59 months. In this analysis, the response variable ‘no’ was recoded as 0 if the child was not malnourished and 1 if the child was malnourished:

4 $$\kern0.5em \log e\ \left[P\left( Yi=1|\ Xi\right)/1-P\left( Yi=1|\ Xi\right)\right]={\log}_e\left[\pi |1-\pi \right]=\alpha +\beta 1 Xi1,\dots, \beta k Xi k$$

Where *Y*_*i*_ is the binary response variable; *X*_*i*_ is the set of explanatory variables, such as sociodemographic characteristics as mentioned in the case of the multinomial model; and *β*_1_,. .., *β*_*k*_ are the coefficients of the *X*_*i*_ variables.

All the analyses have used appropriate sampling weights, which PDHS has provided to account for the survey design using STATA version 16.0. All methods were performed in accordance with the relevant guidelines and regulations.

## Results

Table 1 depicts the percentage distribution of nutritional status of children by various background characteristics. Severe stunting increased with an increase in a child’s age, whereas severe wasting decreased with a child’s age. Only 9% of the children aged 0–12 months were severely stunted compared to almost 19% of children aged 49–60 months. A higher proportion of children in rural areas were severely stunted (19.6% vs. 12.5%), severe wasted (2.4% vs. 2.2%), and severe underweight (9.4% vs. 6%) than their urban counterparts. Similarly, a higher proportion of children whose mothers were uneducated were severely stunted (25.0% vs. 6.9%), severe wasted (2.6% vs. 2.3%), and severe underweight (12.7% vs. 2.2%) than those children whose mother had higher education. Also, a higher proportion of children from the poorest wealth quintile were severely stunted (34.9% vs. 7.7%), wasted (2.6% vs. 1.4%), and underweight (20.2% vs. 3.2%) than children from the richest wealth quintile.

**Table 1:** Percentage distribution of nutritional status of children according to background characteristics in Pakistan

Characteristics	Severe stunt	Moderate stunt	Severe waste	Moderate waste	Severe under weight	Moderate under weight
Age in months						
00–12	8.9	22.3	5.2	26.6	9.4	20.6
13–24	13.2	33.4	2.4	19.4	6.2	17.5
25–36	22.7	47.1	1.6	16.8	8.8	26.4
37–48	23.4	47.2	1.5	16.1	8.0	25.1
49–60	18.6	38.9	0.6	16.1	8.8	25.5
Place of residence						
Urban	12.5	31.5	2.2	6.6	6.0	19.4
Rural	19.6	40.6	2.4	7.2	9.4	24.7
Educational attainment mother						
No Education	25.0	47.7	2.6	8.8	12.7	31.7
Primary	13.9	38.7	2.8	5.3	5.7	19.5
Secondary	9.2	28.3	1.4	5.6	4.0	15.1
Higher	6.9	16.2	2.3	5.1	2.2	8.5
Source of drinking water						
Piped water	13.0	33.4	2.0	8.0	6.3	22.2
Tube well/borewell	17.5	38.1	1.8	6.0	9.0	22.7
Protected well	31.9	55.8	5.8	12.6	7.8	34.5
Unprotected well	29.0	59.0	7.2	12.8	22.5	46.4
River/dam/springs	32.0	53.9	7.3	13.0	15.6	36.3
Others	17.6	34.2	3.0	6.5	4.5	16.1
Type of fuel used for cooking						
Clean	10.4	30.3	2.0	7.0	4.7	17.6
Wood	22.0	41.8	2.4	6.4	11.0	26.1
Crop residual	17.4	49.2	0.0	4.9	7.7	25.7
Animal dung	34.0	51.4	4.3	15.1	23.7	51.3
Others	23.7	45.0	4.5	8.6	7.7	23.2
Current marital status of mother						
Currently married	17.3	37.8	2.3	7.0	8.3	23.1
Others	10.5	21.4	0.9	6.9	5.8	11.8
Sex of household H						
Male	17.4	38.2	2.4	7.4	8.8	24.0
Female	15.8	32.9	1.3	3.5	3.5	14.1
Type of toilet facilities						
Flush toilet	13.0	33.2	2.3	6.7	5.9	19.1
Pit latrine	33.7	53.1	4.0	9.7	18.4	42.8
Open	31.2	52.2	2.0	7.7	19.2	37.7
Other	28.2	50.6	2.4	6.8	4.8	22.2
BMI of mothers						
Underweight	25.6	45.6	2.3	9.9	16.6	37.1
Normal	21.7	43.1	3.2	9.2	9.8	26.2
Overweight	16.8	34.0	3.0	7.0	6.8	19.5
Obese	12.1	29.5	3.5	6.4	4.0	14.4
Wealth quintile						
Poorest	34.9	56.1	2.6	9.5	20.2	41.0
Poorer	19.2	44.9	4.1	9.4	9.2	27.4
Middle	10.9	31.2	1.4	4.8	3.2	14.8
Richer	12.1	30.8	1.9	7.2	4.4	18.0
Richest	7.7	22.9	1.4	3.6	3.2	11.4
Total	17.2	37.6	2.3	7.0	8.3	22.9

Table 2 depicts the percentage distribution of the body-mass index of women by various background characteristics. Results found that obesity increased with age, educational status, and wealth index. Only 4% of the women aged 15–19 years were obese compared to almost 30% of women aged 45–49 years. A higher proportion of rural women (9.5% vs. 5.5%) were underweight than urban women, whereas a higher proportion of urban women were obese (24.3% vs. 19.0%) than rural women. Almost one in every ten uneducated women (11.1%) was underweight, whereas almost one in every six uneducated women (15.7%) was obese. Almost one-fifth (19.5%) of the poorest women were underweight, and only 3% of the richest women were underweight. In contrast, around 7% of the poorest women were obese, and almost 31% of the richest women were obese.

**Table 2:** Percentage distribution of women by body mass index according to background characteristics in Pakistan

Characteristics	Under weight % [95% CI]	Normal % [95% CI]	Over weight % [95% CI]	Obese % [95% CI]	Total No. of Women
Age of mothers					
15–19	14.0 [9.5–18.5]	59.9 [53.5–66.3]	21.8 [16.4–27.2]	4.3 [1.7–7.0]	193
20–24	13.2 [10.7–15.7]	54.4 [50.3–57.7]	22.5 [19.4–25.5]	10.3 [8.1–12.6]	725
25–29	7.9 [6.2–9.6]	40.5 [37.5–43.8]	33.4 [30.4–36.5]	18.0 [15.5–20.5]	918
30–34	7.3 [5.5–9.1]	40.8 [37.4–44.1]	28.6 [25.7–31.9]	23.1 [20.2–26.0]	856
35–39	6.1 [4.4–7.8]	34.1 [31.4–38.2]	30.6 [27.6–34.2]	28.3 [25.1–31.5]	736
40–44	3.9 [2.2–5.7]	33.3 [29.4–37.9]	36.0 [31.7–40.3]	26.4 [22.5–30.4]	462
45–49	5.5 [3.3–7.6]	29.0 [24.7–33.3]	35.3 [30.8–39.8]	30.1 [25.9–34.5]	454
Place of residence					
Urban	5.5[4.5–6.4]	33.3[31.3–35.3]	36.9[34.9–38.9]	24.3[22.5–26.1]	1654
Rural	9.5[8.3–10.7]	45.4[43.3–47.5]	26.1[24.2–27.9]	19.0[17.4–20.7]	2690
Educational attainment mother					
No education	11.1 [9.8–12.4]	46.4 [44.4–48.5]	26.8 [25.0–28.6]	15.7 [14.2–17.1]	2125
Primary	6.3 [4.4–8.3]	40.1 [36.1–44.1]	28.5 [24.8–32.1]	25.1 [21.6–28.6]	674
Secondary	4.7 [3.2–6.2]	34.0 [30.7–37.3]	33.6 [30.3–36.9]	27.7 [24.6–30.8]	930
Higher	3.7 [2.2–5.2]	32.3 [28.6–36.0]	38.8 [34.9–42.7]	25.1 [21.7–28.6]	615
Source of drinking water					
Piped water	6.5 [5.2–7.7]	37.6 [35.1–40.1]	33.3 [30.9–35.8]	22.6 [20.5–24.8]	1250
Tube well/borewell	8.6 [7.4–9.9]	42.4 [40.2–44.6]	29.6 [27.6–31.6]	19.4 [17.6–21.2]	2336
Protected well	8.7 [4.7–12.6]	41.8 [34.9–48.8]	31.8 [25.3–38.4]	17.7 [12.3–23.1]	84
Unprotected well	25.7 [17.1–34.3]	35.5 [26.1–44.9]	25.5 [16.9–34.1]	13.3 [6.6–20.0]	52
River/dam/springs	10.1 [6.1–14.1]	57.7 [51.2–64.2]	23.6 [18.0–29.2]	8.7 [4.9–12.4]	173
Others	5.5 [3.4–7.6]	35.5 [31.1–39.9]	27.5 [23.4–31.6]	31.5 [27.2–35.7]	449
Type of fuel used for cooking					
Clean	4.4 3.6–5.3]	31.9 29.9–33.9]	36.0 34.0–38.1]	27.6 25.7–29.5]	2128
Wood	11.5 10.0–12.9]	47.8 45.5–50.1]	26.3 24.3–28.4]	14.4 12.8–16.0]	1697
Crop residual	13.5 6.1–20.9]	59.0 48.3–69.6]	18.7 10.3–27.2]	8.8 2.7–14.9]	139
Animal dung	15.8 8.9–22.7]	63.8 54.7–72.9]	10.3 4.5–16.1]	10.1 4.4–15.8]	126
Others	7.1 3.7–10.6]	47.1 40.4–53.9]	23.5 17.8–29.3]	22.2 16.6–27.9]	254
Current marital status of mother					
Currently married	8.0 [7.2–8.8]	40.6 [39.1–42.1]	30.4 [29.0–31.8]	21.0 [19.7–22.2]	4188
Others	7.1 [2.9–11.2]	45.3 [37.2–53.3]	25.0 [17.9–32.0]	22.7 [15.9–29.5]	156
Sex of household H					
Male	8.2 [7.3–9.0]	39.9 [38.4–41.4]	30.5 [29.1–32.0]	21.4 [20.1–22.7]	3821
Female	6.4 [4.1–8.7]	47.4 [42.7–52.1]	28.0 [23.7–32.2]	18.2 [14.5–21.8]	523
Type of toilet facilities					
Flush toilet	5.9[5.1–6.7]	37.1[35.5–38.7]	33.3[31.8–34.9]	23.7[22.3–25.1]	3498
Pit latrine	15.5[10.9–20.0]	56.3[50.1–62.5]	17.0[12.3–21.7]	11.2[7.3–15.2]	200
Open	20.7[17.0–24.4]	58.4[53.9–62.9]	16.6[13.2–20.0]	4.2[2.4–6.1]	468
Other	6.8[3.0–10.7]	49.8[42.3–57.4]	19.1[13.2–25.0]	24.2[17.8–30.7]	179
Wealth quintile					
Poorest	19.5 [16.7–22.2]	57.4 [54.0–60.8]	16.2 [13.7–18.7]	6.9 [5.2–8.7]	740
Poorer	8.6 [6.8–10.5]	50.4 [47.1–53.6]	25.8 [23.0–28.7]	15.2 [12.8–17.5]	866
Middle	6.5 [4.8–8.3]	37.4 [34.0–40.8]	33.0 [29.7–36.3]	23.1 [20.1–26.0]	856
Richer	4.8 [3.4–6.3]	36.4 [33.1–39.6]	32.8 [29.7–36.0]	26.0 [23.0–28.9]	898
Richest	2.8 [1.8–3.8]	26.8 [24.1–29.6]	39.8 [36.7–42.9]	30.5 [27.7–33.4]	984
Total	8.0 [7.2–8.8]	40.8 [39.3–42.3]	30.2 [28.8–31.6]	21.0 [19.8–22.2]	4344

Table 3 depicts the binary logistic odds ratio results for stunting, wasting, and underweight children in Pakistan. The odds of severe stunting (OR = 0.24; C.I. = 0.15–0.37), severe underweight (OR = 0.11; C.I. = 0.05–0.22) were lower among children from the richest wealth quintile than their poorest counterparts. Similarly, the odds of severe wasting (OR = 0.59; C.I. = 0.39–0.91) and severe underweight (OR = 0.66; C.I. = 0.5–0.88) were lower among rural children than their urban counterparts. Furthermore, results noted lower odds of severe stunting (OR = 0.39; C.I. = 0.27–0.57) and severe underweight (OR = 0.56; C.I. = 0.32–0.99) among children whose mothers have higher education than those whose mothers were uneducated.

**Table 3:** Results of binary logistic odds ratio of moderating stunting, wasting and underweight among children age 0–59 month in Pakistan

Characteristics	Severe stunt	Moderate stunt	Severe waste	Moderate waste	Severe under weight	Moderate under weight
Wealth quintile						
Poorest®						
Poorer	0.50***(0.40, 0.64)	0.56***(0.45, 0.69)	1.48(0.86, 2.57)	1.20(0.84, 1.72)	0.55***(0.4, 0.76)	0.58***(0.46, 0.73)
Middle	0.40***(0.29, 0.55)	0.48***(0.37, 0.62)	0.50*(0.23, 1.07)	0.66*(0.42, 1.05)	0.25***(0.16, 0.4)	0.41***(0.31, 0.55)
Richer	0.38***(0.27, 0.55)	0.40***(0.30, 0.54)	0.51(0.22, 1.2)	0.73(0.43, 1.22)	0.27***(0.16, 0.46)	0.41***(0.30, 0.58)
Richest	0.24***(0.15, 0.38)	0.28***(0.20, 0.39)	0.46(0.17, 1.21)	0.39***(0.21, 0.73)	0.12***(0.06, 0.25)	0.21***(0.14, 0.32)
Age of mothers						
15–19®						
20–24	1.58*(0.93, 2.68)	1.24(0.82, 1.87)	1.01(0.38, 2.71)	1.15(0.57, 2.33)	1.09(0.57, 2.06)	0.89(0.57, 1.38)
25–29	1.47(0.87, 2.48)	1.42*(0.95, 2.13)	0.64(0.24, 1.7)	1.10(0.55, 2.21)	0.91(0.48, 1.71)	1.02(0.66, 1.57)
30–34	1.47(0.86, 2.50)	1.30(0.86, 1.96)	0.71(0.26, 1.93)	1.17(0.58, 2.37)	0.90(0.47, 1.72)	1.07(0.69, 1.67)
35–39	1.39(0.80, 2.40)	1.20(0.78, 1.84)	0.50(0.17, 1.46)	0.96(0.46, 2)	0.86(0.44, 1.69)	0.94(0.59, 1.49)
40–44	1.78*(0.95, 3.32)	1.40(0.85, 2.32)	0.90(0.28, 2.92)	1.32(0.57, 3.02)	0.87(0.39, 1.95)	1.33(0.78, 2.28)
45–49	1.24(0.56, 2.76)	0.73(0.37, 1.44)	0.27(0.03, 2.48)	0.55(0.14, 2.11)	0.40(0.12, 1.32)	0.93(0.46, 1.89)
Place of residence						
Urban®						
Rural	0.94(0.76, 1.15)	0.88(0.75, 1.04)	0.58**(0.38, 0.89)	0.66***(0.5, 0.87)	0.65***(0.49, 0.87)	0.80**(0.66, 0.96)
Sex of household Head						
Male®						
Female	0.88(0.66, 1.16)	0.92(0.74, 1.14)	0.67(0.32, 1.40)	0.56**(0.35, 0.89)	0.50***(0.31, 0.81)	0.71**(0.54, 0.92)
Source of drinking						
water						
Piped water®						
Tube well/borewell	0.97(0.79, 1.20)	0.93(0.79, 1.09)	0.93(0.58, 1.48)	0.82(0.61, 1.09)	1.10(0.82, 1.47)	0.91(0.76, 1.10)
Protected well	1.31(0.87, 1.99)	1.23(0.86, 1.76)	3.06***(1.54, 6.07)	2.59***(1.60, 4.19)	1.26(0.70, 2.29)	1.33(0.9, 1.96)
Unprotected well	1.23(0.74, 2.03)	1.42(0.88, 2.31)	2.97**(1.20, 7.36)	1.85*(0.96, 3.59)	2.24***(1.28, 3.91)	1.66**(1.04, 2.66)
River/dam/springs	0.89(0.66, 1.21)	0.75**(0.58, 0.96)	1.04(0.51, 2.08)	1.24(0.82, 1.90)	0.73(0.46, 1.14)	0.77*(0.57, 1.02)
Others	1.44**(1.02, 2.04)	1.08(0.82, 1.43)	1.87*(0.97, 3.58)	1.52*(0.98, 2.36)	2.01***(1.28, 3.16)	1.34*(0.98, 1.84)
Type of fuel used for						
cooking						
Clean®						
Wood	0.98(0.76, 1.27)	0.88(0.72, 1.07)	1.02(0.59, 1.75)	0.86(0.61, 1.21)	0.92(0.64, 1.33)	0.85(0.67, 1.07)
Crop residual	0.81(0.47, 1.39)	0.96(0.6, 1.54)	1.01***(0, 0)	0.64(0.26, 1.59)	0.35**(0.15, 0.84)	0.66(0.39, 1.11)
Animal dung	1.43(0.85, 2.39)	1.03(0.63, 1.66)	1.28(0.40, 4.10)	1.74(0.88, 3.47)	1.30(0.69, 2.45)	1.93***(1.18, 3.15)
Others	0.88(0.56, 1.40)	0.84(0.57, 1.23)	3.23***(1.36, 7.67)	1.85**(1.02, 3.35)	1.07(0.56, 2.04)	1.03(0.68, 1.57)
Type of toilet Facilities						
Flush toilet®						
Pit latrine	1.48**(1.08, 2.02)	1.29*(0.97, 1.73)	0.91(0.44, 1.87)	0.82(0.51, 1.34)	1.17(0.78, 1.77)	1.33*(0.99, 1.80)
Open	1.14(0.86, 1.5)	1.09(0.85, 1.41)	0.83(0.43, 1.61)	1.01(0.66, 1.51)	1.41*(0.99, 1.99)	1.10(0.85, 1.44)
Other	1.56**(1.07, 2.27)	1.74***(1.24, 2.46)	0.14***(0.04, 0.49)	0.37***(0.19, 0.73)	0.51**(0.27, 0.97)	0.88(0.60, 1.28)
Current marital status						
Never in union®						
Ever married	0.92(0.40, 2.14)	0.75(0.39, 1.45)	0.83(0.11, 6.23)	1.23(0.43, 3.53)	0.94(0.28, 3.20)	0.89(0.42, 1.90)
Educational attainment						
No education®						
Primary	0.68***(0.52, 0.88)	0.77**(0.63, 0.95)	0.81(0.46, 1.42)	0.60**(0.41, 0.90)	0.64**(0.43, 0.95)	0.77**(0.61, 0.98)
Secondary	0.51***(0.39, 0.67)	0.64***(0.52, 0.78)	0.54*(0.29, 1.00)	0.74*(0.52, 1.05)	0.61**(0.40, 0.91)	0.62***(0.49, 0.79)
Higher	0.37***(0.25, 0.55)	0.40***(0.31, 0.52)	0.63(0.29, 1.37)	0.68(0.43, 1.08)	0.55**(0.31, 0.98)	0.40***(0.28, 0.56)
BMI of Mothers						
Underweight						
Normal	0.97(0.73, 1.28)	1.03(0.81, 1.32)	1.50(0.70, 3.21)	0.98(0.66, 1.45)	0.71**(0.51, 0.99)	0.69***(0.54, 0.89)
Overweight	0.95(0.69, 1.29)	0.88(0.67, 1.14)	1.54(0.69, 3.44)	0.76(0.49, 1.18)	0.62**(0.42, 0.92)	0.58***(0.44, 0.77)
Obese	0.64**(0.45, 0.92)	0.70**(0.53, 0.94)	1.92(0.82, 4.48)	0.69(0.42, 1.13)	0.38***(0.23, 0.62)	0.40***(0.29, 0.55)

CI-95%, Significance ***p < .001., ** *p* < 0.01, *p < 0.05

Table 4 depicts the relative risk ratio of BMI among women in Pakistan by various background characteristics. The results found that the RRR of being overweight (RRR = 3.32; C.I. = 2.14–5.17) and obese (RRR = 15.86; C.I. = 7.42–33.91) than normal BMI was higher among women aged 45–49 years of age than women aged 15–19 years of age. The RRR of being underweight (RRR = 0.72; C.I. = 0.51–1.01) than normal BMI was lower among rural women than urban women. In contrast, the RRR of being obese (RRR = 1.46; C.I. = 1.17–1.82) than normal BMI was higher among rural women than urban women. The RRR of being underweight decreased with an increase in household wealth quintile, whereas the RRR of being overweight and obese increased with an increase in household wealth quintile. The results found that the RRR of being underweight (RRR = 0.38; C.I. = 0.20–0.75) than normal BMI was lower among women from the richest wealth quintile than women who belonged to the poorest wealth quintile. Furthermore, the RRR of being overweight (RRR = 3.70; C.I. = 2.47–5.54) and Obese (RRR = 4.35; C.I. = 2.67–7.07) than normal BMI were higher among women from richest wealth quintile than women belonged to poorest wealth quintile.

**Table 4:** Results of multinomial logistic regression relative risk ratio of BMI among women in Pakistan

Characteristics	Under-weight RRR (95% CI)	Overweight RRR (95% CI)	Obese RRR (95% CI)
Age of mothers			
15–19®			
20–24	1.10(0.68,1.80)	1.06(0.70,1.60)	2.42**(1.14,5.16)
25–29	0.85(0.51,1.40)	2.24***(1.50,3.34)	6.05***(2.89,12.66)
30–34	0.71(0.43,1.19)	1.90***(1.27,2.85)	8.13***(3.89,16.98)
35–39	0.70(0.41,1.20)	2.45***(1.63,3.70)	11.93***(5.69,24.97)
40–44	0.48**(0.25,0.92)	2.94***(1.91,4.54)	12.23***(5.74,26.08)
45–49	0.78(0.42,1.44)	3.32***(2.14,5.17)	15.86***(7.42,33.91)
Place of residence			
Urban®			
Rural	0.72*(0.51,1.01)	0.88(0.72,1.08)	1.46***(1.17,1.82)
Sex of household H			
Male®			
Female	0.71*(0.48,1.06)	0.80*(0.63,1.01)	0.70**(0.54,0.92)
Source of drinking water			
Piped water®			
Tubewell/borewell	1.02(0.76,1.38)	1.03(0.86,1.23)	0.98(0.80,1.2)
Protected well	1.10(0.46,2.59)	1.33(0.76,2.32)	1.17(0.60,2.30)
Unprotected well	2.43**(1.12,5.25)	2.35**(1.10,5.02)	2.27*(0.88,5.89)
River/dam/springs	0.71(0.40,1.29)	0.97(0.64,1.48)	0.64(0.35,1.16)
Others	1.26(0.72,2.20)	0.86(0.63,1.19)	1.34*(0.96,1.86)
Type of fuel used for cooking			
Clean®			
Wood	1.11(0.75,1.64)	0.98(0.78,1.24)	0.72**(0.56,0.94)
Crop residual	0.98(0.52,1.86)	0.66(0.40,1.09)	0.40***(0.21,0.78)
Animal dung	1.04(0.55,1.95)	0.34***(0.18,0.64)	0.47**(0.24,0.92)
Others	0.92(0.47,1.82)	0.94(0.61,1.45)	0.75(0.45,1.26)
Type of toilet Facilities			
Flush toilet®			
Pit laterine	1.19(0.75,1.91)	0.60**(0.39,0.92)	0.69(0.41,1.14)
Open	1.50**(1.05,2.16)	0.72*(0.51,1.00)	0.32***(0.19,0.54)
Other	0.75(0.34,1.64)	0.62*(0.36,1.08)	1.07(0.59,1.94)
Current marital status			
Never in union®			
Currently married			
Others	1.03(0.52,2.01)	0.64**(0.42,0.98)	0.78(0.5,1.22)
Educational attainment			
No education®			
Primary	0.83(0.57,1.21)	0.99(0.79,1.25)	1.36**(1.05,1.76)
Secondary	0.83(0.54,1.26)	1.06(0.84,1.33)	1.46***(1.12,1.89)
Higher	0.77(0.45,1.35)	1.08(0.82,1.42)	1.04(0.76,1.42)
Wealth quintile			
Poorest®			
Poorer	0.61***(0.43,0.88)	1.64***(1.21,2.21)	1.70***(1.14,2.52)
Middle	0.66*(0.42,1.03)	2.46***(1.76,3.43)	2.71***(1.78,4.13)
Richer	0.50**(0.29,0.86)	2.43***(1.68,3.52)	3.06***(1.94,4.80)
Richest	0.38***(0.2,0.75)	3.70***(2.47,5.54)	4.35***(2.67,7.07)

CI-95%, Significance ***p < .001., ** p < 0.01, *p < 0.05; Base category for multinomial is normal BMI; RRR (95% CI) if relative risk ratio and 95% confidence interval

## Discussion

This research paper investigated the factors associated with the nutritional status of mothers and their children. The three indicators, namely; stunting, wasting, and underweight, were categorized to examine nutritional status among children, whereas nutritional status among mothers was categorized by body-mass index. The study has revealed that almost one-sixth (17.2%) of the children were severely stunted, another 2 % were severely wasted, and almost 8% were severely underweight. Furthermore, the results revealed that the risk of severe stunting was higher among the poorest children and children whose mothers were uneducated. Similarly, severe wasting was higher among urban children. The risk of severe underweight was higher among the poorest children, urban children, male children, children defecating in the open, and children whose mothers had no education.

### Nutritional status among children

Corroborating with previous findings [1, 25–28], this study noted the reduced risk of severe stunting among children whose mothers had higher education. In a few studies, maternal education was associated with greater reductions in the odds of stunting among children than paternal education [28, 29]. Mothers are primary caregivers for the children, and therefore, their education level can have a direct and substantial impact on child stunting than that of fathers [28]. Lower levels of education among mothers could result in limited family income and has consequences on individual care and attention given to the child [26]. Furthermore, educated mothers are more likely to be conscious about their child’s health, improving stunting levels [30].

The study further noted higher odds of stunting among children from the poorest households. Several previous literature pieces agree with these findings [1, 26, 29, 31–33]. The effect of increasing wealth on reducing stunting could be explained by the increasing purchasing capacity that promotes and protects children’s health. Several studies have examined an association between low income and malnutrition, often leading to stunting [34, 35]. Children from poor households tend to have limited access to food and healthcare services, making them more vulnerable to growth failure [36].

Unlike previous studies [1], this study failed to find a concrete association between wasting and maternal education and household wealth. Results found that the odds of severe wasting were lower among rural children and higher among children drinking water from protected or unprotected well. Deviating from this finding, a study conducted in six Asian countries, including Pakistan, noted a higher risk of wasting among rural children [37]. Future studies should explore why the wasting is lower among rural children in Pakistan as several previous studies have noted an otherwise finding in Pakistan [38]. Being an acute form of malnutrition, wasting could partially explain the higher odds of wasting among urban children in this study. It is suggested to carry out further study to examine the determinants of rural-urban differential in wasting in Pakistan. Countering urban-rural differential in 15 sub-Saharan African countries, a study noted narrowing urban-rural differential in child undernutrition due to an increase in undernutrition in urban areas [39].

In agreement with previous studies, this study noted that drinking water from well and other sources lead to higher odds of wasting than drinking piped water [37, 40, 41]. Exploring the social context of wasting across regions, a study showed that access to safe water is a crucial determinant of wasting in Asian countries [42]. Drinking water from unimproved sources may cause diarrhea which could further lead to wasting among children [40]. No matter, drinking water from protected wells also led to higher odds of wasting among children in this study. People use groundwater without any treatment, leading to diarrhea and further wasting among children in Pakistan [40]. Since wasting is an acute form of malnutrition, it is easily manipulated by diarrheal conditions in Pakistan [40]. Diarrhea results in poor digestion, malabsorption, and lower appetite leading to short-term acute malnutrition, also known as wasting among children [43].

In agreement with an existing scholarship [1, 44], this study noted reduced odds of underweight among children with increased household wealth. Low household economic status can contribute significantly to the poor nutritional status of mothers, which further has a bearing on nutritional outcomes among children [45, 46]. Agreeing with previously available literature [1, 25, 29, 36, 44, 47, 48], the findings from this study noted reduced odds of underweight among children with an increase in maternal educational status. Educated mothers are well-informed about the nutritional choices for their children and prefer to follow hygiene and sanitation practices leading to a reduced risk of being underweight among children [1]. To add more, educated mothers make better choices of available health services for improved healthcare of their children [44].

### Nutritional status among women

Other than examining the children’s nutritional status, this article also examines the nutritional status of the women, albeit with a different indicator. Body-mass index indicator was used to assess the women’s nutritional status and examine the predictors of nutritional status among women in Pakistan. In line with previous findings, the study noted a higher odds of overweight and obesity among women at higher ages [49–52]. Parity increases with an increase in age and could be a plausible factor of higher obesity among women [53, 54]. Another plausible reason for obesity among women at high age is that women at older ages are more likely to remain physically inactive and consume more energy-dense food leading to obesity [55]. Another plausible reason could be the change in body composition due to increased age, leading to increased body fat mass [56].

The odds of being obese were higher among educated women than their uneducated counterparts, similar to the previous study finding [57–59]. A plausible fact is that educated women are less involved in physical activity leading to a higher risk of obesity [60]. In addition, women with high education levels tend to have better financial status than uneducated women [61], which is further linked to higher odds of obesity among women [45, 46]. Similar results were also noted in this study, where higher odds of overweight and obesity among women from the richest wealth quintile were observed. Several previous studies also reported a higher risk of obesity among rich women [58, 62, 63]. The high risk of obesity among rich women could be attributed to the notion that rich people follow a sedentary lifestyle and are engaged in less labour-intensive work [64]. Moreover, rich people tend to consume more energy due to a greater purchasing capacity leading to obesity [60]. In addition, affluent women are more likely to follow a sedentary lifestyle such as viewing television, further linked to higher obesity [65].

The results found that the odds of being underweight were lower among rural women, and odds of obesity were higher among rural women. This finding deviates from several previous findings where the risk of obesity was higher among urban women [50, 66, 67]. The rural areas are also experiencing nutritional transition, and people prefer fast foods and other energy-dense food [59]; all this could have led to higher obesity risk among rural women. Furthermore, a multi-country study has confirmed that over the three decades from 1985 to 2017, a large share of the increase in obesity worldwide is attributed to the rise in obesity in rural areas [68]. People in rural areas fast adopt the lifestyle followed in urban areas and are more vulnerable and susceptible to chronic illnesses, including obesity [69]. The high prevalence of obesity may be due to eating more carbohydrates and fats-rich diets like bread and rice [70], which could be attributed to a higher risk of obesity among rural women. At first, not much literature indicates the rise of obesity among rural women. It has been noted that urban women had higher levels of obesity than rural women. This is quite an interesting and relatively new one; therefore, this finding requires an in-depth exploration, and further studies are warranted to explore this phenomenon.

## Strength and limitation of the study

This study utilized data from a nationally representative survey, giving nationally-comparable estimates. One key limitation, however, was that we could not establish the cause and effect relationships; because of the cross-sectional nature of the study design.

## Conclusion

This study has highlighted determinants associated with maternal and child nutritional status, whereby a child’s nutritional status was measured by stunting, wasting, and underweight, and the mother’s nutritional status was measured by BMI. The main risk factors for child’s poor nutritional status include low household wealth, urban residence, and mother’s educational status. Similarly, the main risk factors for women’s poor nutritional status include increasing age of the women, educational status of the women, rural residence, and household wealth. The findings indicate the need for interventions to improve nutritional status among children and women. Emphasis should be placed on providing relevant health education among mothers as it would not only improve their nutritional status but also improve their child’s nutritional status simultaneously. Furthermore, policies focusing on poverty alleviation and improving the nutritional status of poorer sections of society are needed to address the nutritional disadvantages among women and children belonging to poor households.

## Data Availability

The datasets generated and/or analysed during the current study are available with DHS program. The data can be requested at: https://dhsprogram.com/data/available-datasets.cfm

## Abbreviations

BMI: Body Mass Index
CI: Confidence Interval
CIDA: Canadian International Development Agency
EPI: Expanded Program of Immunization
FP: Family Planning
IMR: Infant Mortality Rate
LHWs: Lady Health Workers
MI: Micronutrient Initiative
MNCH: Maternal and Child Health
OR: Odds Ratio
PDHS: Pakistan Demographic Health Survey
RRR: Relative Risk Ratio
UNICEF: United Nations. Children Fund
